# Recent Technologies for Transcutaneous Oxygen and Carbon Dioxide Monitoring

**DOI:** 10.3390/diagnostics14080785

**Published:** 2024-04-09

**Authors:** Sara Bernasconi, Alessandra Angelucci, Anastasia De Cesari, Aurora Masotti, Maurizio Pandocchi, Francesca Vacca, Xin Zhao, Chiara Paganelli, Andrea Aliverti

**Affiliations:** Dipartimento di Elettronica, Informazione e Bioingegneria, Politecnico di Milano, 20133 Milan, Italy; sara.bernasconi@polimi.it (S.B.); alessandra.angelucci@polimi.it (A.A.); anastasia.decesari@mail.polimi.it (A.D.C.); aurora.masotti@mail.polimi.it (A.M.); maurizio.pandocchi@mail.polimi.it (M.P.); francesca.vacca@mail.polimi.it (F.V.); xin1.zhao@mail.polimi.it (X.Z.); chiara.paganelli@polimi.it (C.P.)

**Keywords:** continuous monitoring, non-invasive monitoring, transcutaneous O_2_ monitoring, transcutaneous CO_2_ monitoring, wearable measurement systems

## Abstract

The measurement of partial pressures of oxygen (O_2_) and carbon dioxide (CO_2_) is fundamental for evaluating a patient’s conditions in clinical practice. There are many ways to retrieve O_2_/CO_2_ partial pressures and concentrations. Arterial blood gas (ABG) analysis is the gold standard technique for such a purpose, but it is invasive, intermittent, and potentially painful. Among all the alternative methods for gas monitoring, non-invasive transcutaneous O_2_ and CO_2_ monitoring has been emerging since the 1970s, being able to overcome the main drawbacks of ABG analysis. Clark and Severinghaus electrodes enabled the breakthrough for transcutaneous O_2_ and CO_2_ monitoring, respectively, and in the last twenty years, many innovations have been introduced as alternatives to overcome their limitations. This review reports the most recent solutions for transcutaneous O_2_ and CO_2_ monitoring, with a particular consideration for wearable measurement systems. Luminescence-based electronic paramagnetic resonance and photoacoustic sensors are investigated. Optical sensors appear to be the most promising, giving fast and accurate measurements without the need for frequent calibrations and being suitable for integration into wearable measurement systems.

## 1. Introduction

Today’s digital healthcare systems aim at providing clinical professionals with the ability to continuously and safely monitor the vital signs of patients at risk, granting both measurement accuracy and comfort for the patient [1]. This perspective is facilitated by the possibility to remotely monitor the patient, with a consequently improved quality of care, freedom outside the hospital, the design of treatments tailored to the patient, and reduced costs [1].

Respiratory status, representing the state and operation of an individual’s respiratory system, assumes paramount significance in the evaluation of human well-being. Consequently, it receives substantial clinical attention, and the pivotal parameters employed in its appraisal encompass the respiratory rate (RR), partial pressures of oxygen (PO_2_) and carbon dioxide (PCO_2_), and blood oxygen saturation (SaO_2_) [1,2].

Oxygen (O_2_) and carbon dioxide (CO_2_) partial pressures, also known as tensions, are the target quantities for transcutaneous monitoring. They describe how well the lungs and the bloodstream exchange gases [2] and should be continuously monitored in patients whose respiratory vital signs may change frequently and rapidly [3]. Extremes in these parameters account for hyperoxia and hypoxia, and hypercapnia and hypocapnia [4].

Continuous monitoring could prevent the occurrence of certain pathological conditions related to the respiratory system and beyond, such as retinopathy of prematurity (ROP) [1,4,5]. Furthermore, considering that symptoms of respiratory failure become apparent only after tissue damage has occurred, the real-time monitoring of PCO_2_ and PO_2_ assumes a crucial significance, as it can detect deteriorations in clinical conditions at an earlier stage, as in the case of PO_2_ in COVID-19 patients [6].

Given the importance of these parameters, in recent years, extensive research endeavors have been undertaken in the substitution of state-of-the-art bulky devices towards miniaturized and wearable devices for transcutaneous monitoring, which is particularly advantageous from many standpoints, as will be illustrated in the present work.

The transcutaneous technique was originally introduced in the Intensive Care Unit (ICU), especially for neonates, where it offered additional advantages such as early discharge to a more stable home life and a reduced risk of undiagnosed issues [1]. Then, it spread into a variety of medical applications, such as adult ICU [7], mechanical ventilation, anesthesia, bronchoscopy, sleep studies, pulmonary stress, and respiratory research [8].

Section 2 describes the physiology underlying the measurement of the partial pressure of blood gases. In Section 3, the gold standard and commercially available technologies will be outlined. Then, recent technologies’ necessity and challenges in regard to their respective implementations are reported in Section 4, Section 5 and Section 6 for, respectively, oxygen, carbon dioxide, and combined sensing. Section 7 provides a discussion, while Section 8 encapsulates the conclusions.

The literature review extended until September 2023, and it was conducted using the online archives Pubmed, Google scholar, IEEE, SPIE digital library, Espacenet, and Google patents. The used keywords were “Carbon dioxide”, “continuous monitoring”, “non-invasive monitoring”, “transcutaneous CO_2_ monitoring”, “transcutaneous monitoring”, “transcutaneous O_2_ monitoring”, “oxygen”, and “wearable”. Peer-reviewed papers, patents, guidelines, websites, datasheets, and manuals were included in the review.

## 2. Physiology

Transcutaneous monitoring exploits the ability of oxygen and carbon dioxide to diffuse through the skin [9], due to the gas gradients between the human body and the atmosphere.

In 1851, Von Gerlach illustrated the respiratory activity of superficial capillary blood in the skin. Through a 24 h placement of an air-filled chamber, crafted from a horse bladder, on his chest, Von Gerlach noted a rise in the chamber’s CO_2_ concentration from 0 to 2.5 percent, accompanied by a decline in the O_2_ concentration from 21 to 19 percent. This phenomenon is associated with internal respiration, also known as cellular respiration, contributing from approximately 2 to 3% to the overall gas exchange between the human body with its surroundings.

Gases undergo diffusion through the skin, which is composed of two main layers, the superficial one, the epidermis, and the inner one, the dermis, where a dense network of capillaries is organized in vertical structures of about 0.2–0.4 mm [10]. The diffusion speed of blood gases from blood vessels through the skin to the environment can be influenced by externally induced phenomena, like heating (addressed later in this section), and by multiple patient factors, including skin thickness, especially the stratum corneum in the epidermis [11], blood vessel reactivity, and arterial gas concentrations [9,12].

The assessment of the transcutaneous partial pressure of gases is an indirect method [13] and serves as a surrogate for the arterial partial pressure. Nevertheless, in certain scenarios, this transcutaneous assessment can yield valuable insights that extend beyond the information provided by arterial partial pressures. In fact, the transcutaneous pressure derives both from the blood and from cells’ metabolism [14], which depends on an adequate supply of O_2_ and an appropriate amount of CO_2_ to maintain an acid-base balance [15]. Thus, transcutaneous sensing can give information either on abnormal ventilation [1] or the perfusion of tissues.

Considering the details of each of the two gases, the arterial partial pressure of oxygen in the blood is a valid index of arterial oxygenation, directly proportional to the concentration of dissolved oxygen in the plasma, as dictated by Henry’s Law. Thanks to this ‘driving force’, oxygen reaches the mitochondria in tissues and enables cellular respiration with the creation of ATP [16]. The concentration of oxygen in the arterial blood is determined by the partial pressure of ambient air, the adequacy of gas exchange and ventilation, and the concentration of hemoglobin and its affinity to oxygen. The arterial partial pressure of oxygen is also related to the oxygen saturation, expressing the percentage of binding sites of hemoglobin occupied by oxygen through the oxygen–hemoglobin dissociation curve.

Oxygen saturation is another important parameter for assessing respiratory status and can be non-invasively measured through pulse oximetry (described in Section 3.3).

Concerning CO_2_, PtCO_2_ can give important clinical information about the hemodynamic status of patients [17], since it depends on the CO_2_ produced by the tissue, the removed CO_2_ from the tissue by perfusion, and the reference value of CO_2_ at the tissue inlet represented by the arterial CO_2_ content. Hence, two different approaches can be carried out, one focused on the respiratory area, where PtCO_2_ is used to non-invasively estimate the arterial CO_2_ partial pressure (PaCO_2_), the other on the cardio-vascular area, where PtCO_2_ can be representative of a possible hemodynamic failure in a patient [17].

Ranges for the partial pressures of oxygen and carbon dioxide in healthy humans are, respectively, 75–100 mmHg [18] and 35–45 mmHg (both for adults [3] and newborns [17]), and shifts in physiological values may cause or may be a cause of pathological conditions.

A deviation in the arterial partial pressure of oxygen results in the conditions known as hypoxia and hyperoxia. The former occurs because of various pathological mechanisms. Key factors contributing to this decline encompass diminished inhalation of oxygen, for instance due to altitudes, hypoventilation, diffusion limitations, and a ventilation/perfusion mismatch (V/Q mismatch) [19]. On the other hand, a very high arterial PO_2_, hyperoxia, may reduce oxygen delivery to tissues due to a vasoconstriction or reduction in cardiac output [2,20,21].

In regard to the carbon dioxide arterial partial pressure, its variations are called hypocapnia and hypercapnia. More specifically, hypocapnia is a deficiency of CO_2_ in the organism, and its principal physiologic causes are related to hyperventilation [22]. Other causes are hypoxemia, pulmonary disorders, cardiovascular disorders, or central nervous system disorders. On the contrary, hypercapnia is an increase in arterial PCO_2_, and can be determined by hypoventilation or by increasing ventilation/perfusion inequality, affecting the arterial PO_2_, which will fall, and the arterial PCO_2_, which will rise, or by breathing in a room with an elevated concentration of CO_2_.

In patients with cardiopulmonary diseases, hypocapnia, associated with a mismatch between ventilation and perfusion, can be severe enough to be fatal, whereas hypoventilation in a healthy patient could lead to a traumatic injury to any portion of the respiratory system. Therefore, PtCO_2_ measurement is more suitable than repeated arterial blood sampling to reflect CO_2_ dynamics, ventilation/perfusion mismatch, and blood flow and detect these anomalies [23]. For instance, in the case of hemodynamic failure, a mismatch between PtCO_2_ and PaCO_2_ occurs, called the transcutaneous–arterial PCO_2_ gap, leading to tissue hypercarbia uncorrelated with PaCO_2_. By measuring this gap, the systemic and local cutaneous perfusion condition of the patient can be evaluated [17]. The agreement between PaCO_2_ and PtCO_2_ is influenced also by the body location of the measure. An interesting meta-analysis [9] demonstrated that, for CO_2_, the highest accordance between transcutaneous and arterial partial pressure is found when positioning the sensor at the earlobe.

It must be noted that the reliability of this measurement, independent from the technology used, may be compromised in individuals, especially infants, with impaired perfusion, acidosis [24], oedema, or those receiving vasoconstrictor medications [25].

### 2.1. Heating

The topic of heating is of primary importance when dealing with transcutaneous sensing.

Due to the large mass transfer resistance of human skin, which is the resistance opposed to the diffusion of gases, transcutaneous monitoring requires a considerable amount of time to become stable when moved from ambient air to human skin. Utilizing a heating system led to a reduction in stabilization time to 20 min, contrasting starkly with the over 2 h duration required in the absence of heating [2,15,26,27].

Typically, heating the skin to a range between 37 °C and 45 °C makes the skin blood flow increase by three to four times, enhancing the arterial blood contribution by opening the precapillary sphincter arterioles, a phenomenon known as arterialization [17]. As a consequence, the diffusion of blood gases and the delivery of blood beneath the sensor increase [14,17,26] by the means permeability improvements in the arterioles and capillaries [14,17]. Furthermore, local warming is used to normalize the blood flow and is a strategic approach to standardizing the conditions around the monitoring site, allowing for more reliable and consistent transcutaneous measurements.

It must be noted that increasing the skin temperature also determines an increase in the metabolism of the cells. Consequently, there is an excess of CO_2_ [14,28] and an increased consumption of O_2_. An increase in PCO_2_ by 4.5%/°C [8] induces tissue hypercarbia [17], so the interpretation of the measured value must consider the operating conditions in terms of heating [17] through the application of a correction factor [8].

Regarding oxygen, a rise in temperature causes a shift in the oxygen dissociation curve, reducing the binding of O_2_ to hemoglobin, thus, making more oxygen accessible to cells. This phenomenon, together with increased diffusion, results in an increase in O_2_ [7]. Nonetheless, increased cellular metabolism brings about local tissue oxygen consumption, partially offsetting the rise in O_2_ [29,30]. A patent published in 2010 [31] attempted to formulate a correction factor for both PtCO_2_ and PtO_2_ which comprehended the reference temperature, usually 37 °C, the local skin temperature, and a factor which is related to the local blood flow in proximity to the skin.

Warming the skin also improves the SpO_2_ measurement performance, increasing the arterial pulsation signal and reducing the non-arterialized tissue component signal [32].

Skin warming has an important role in transcutaneous measurements also because it improves the correlation between arterial partial pressure and transcutaneous partial pressure [14,17,31], increases sensitivity to low levels of oxygen [7], and stabilizes noise fluctuations [32]. Accordingly, the temperatures that are found in the literature are 40–42 °C for PtCO_2_ [10,26,32,33], with 44 °C as the threshold not to be exceeded [34], and 43–44 °C for PtO_2_ [10], with 45 °C being the maximum tolerable temperature [35]. The slightly higher temperature to be applied for O_2_ sensing is due to its lower diffusion capacity and permeability with respect to CO_2_.

The heating application carries some problems that makes its removal from transcutaneous sensing devices desirable. First, it can cause discomfort in patients [3], even posing the risk of skin burn wounds [34], especially in patients with sensitive and delicate skin such as neonates [4,36]. Lowering the temperature may represent a potential compromise, acknowledging a trade-off with accuracy [37]. To maintain the best possible accuracy, the sensor should be frequently repositioned (every three to four hours, down to every hour) with consequent re-calibration [4,8,36].

Furthermore, there are issues undermining the potential use of heating units in a wearable sensor, such as its high power consumption [3,34] and the complexities associated with integrating a heating element into a miniaturized device [18].

To avoid the problem of heating, since the 1980s, alternative indices for assessing the respiratory status of patients have been explored, such as oxygen saturation through pulse oximetry [4], aiming at combining the advantages of non-invasive sensing with the additional benefit of not requiring heating. Nevertheless, the informational content derived from a transcutaneous measurement surpasses that obtained solely from oxygen saturation. In fact, saturation relies on the presence of perfusion and does not provide information about the dissolved oxygen within the tissues and cells. For instance, despite a normal SpO_2_ value, hypoxia and ischemia may still occur, conditions that can only be detected through the measurement of dissolved oxygen [38].

## 3. Overview of Available Techniques

### 3.1. Arterial Blood Gas Analysis

An arterial blood gas (ABG) analysis is a medical examination that involves the collection of an arterial blood sample and measures several critical parameters in the blood, including oxygen and carbon dioxide tensions, arterial saturation, blood acidity (pH), and bicarbonate levels.

An ABG analysis provides valuable insights into a person’s respiratory and metabolic health. It helps healthcare professionals to assess oxygenation, i.e., how well the lungs are oxygenating the blood, ventilation, i.e., how effectively the lungs are eliminating carbon dioxide, and the body’s acid-base balance. This information is crucial for diagnosing and managing various respiratory and metabolic disorders, such as chronic obstructive pulmonary disease (COPD).

This technique represents the gold standard against which newly developed sensors and methodologies are validated [10], as it provides the most accurate measurements of PO_2_ and PCO_2_ [39]. However, it is invasive and, therefore, may lead to complications and local reactions of the body such as pain, inflammation, infection, and tissue and nerve damage [9,15,35,36,40,41]. Furthermore, because of the inherent characteristics of this method, it offers only a snapshot measurement [36,39,42] and does not allow for continuous monitoring, a crucial aspect in situations characterized by rapid variations in respiratory parameters. In addition, clinical infrastructures and trained personnel are required for the collection and analysis of arterial blood samples [43].

### 3.2. End-Tidal Monitoring

End-tidal monitoring provides real-time [44] measurements, evaluating the partial pressure or concentration of carbon dioxide at the end of every exhalation of the patient. While this technique is predominantly employed for assessing CO_2_, it is noteworthy that O_2_ in inspired/expired air also serves as a sensitive and valuable indicator of adequate ventilation and appropriate oxygen supply [45].

The values of exhaled PCO_2_ are a proxy of the alveolar PCO_2_ in normal conditions, as the PetCO_2_ measurement, the highest CO_2_ concentration in the exhaled breath, originates from the alveoli [46].

PetCO_2_ has been historically used during general anesthesia and is progressively becoming a common practice in other clinical fields like emergency environments and critical care [44]. It is used for many applications like cardiopulmonary resuscitation, airway assessment, sedation, and analgesia [47]. It can be useful for the early recognition of potential breathing complications and the recognition of respiratory compromise or failure [44,48], allowing for prompt diagnosis and, consequently, a timely intervention or correction.

End-tidal measurement is commonly said to be a non-invasive technique [1,8,42,49,50]. A capnograph is the medical device used to perform this assessment, exploiting the principle of spectrophotometric absorption for CO_2_ detection. Instead, for oxygen in modern anesthetic machines, paramagnetic systems are generally used.

Even if it is a consolidated technique, its accuracy is compromised both in intubated [14,39] and non-intubated patients [1,8], and many studies suggest that PtCO_2_ is preferred over PetCO_2_ [8], with the former being more accurate [14,51]. In addition, certain situations may affect the reliability of this measurement. For instance, an elevated breathing frequency may exceed the capnograph’s response capabilities, and the use of filters between the patient airway and the capnograph’s sampling line may lead to artificially low EtCO_2_ readings [52].

### 3.3. Pulse Oximetry

While the arterial saturation of oxygen is traditionally evaluated through an arterial blood gas (ABG) analysis, peripheral saturation (SpO_2_) serves as a reliable proxy for arterial oxygen saturation (SaO_2_). Notably, SpO_2_ can be non-invasively measured using photoplethysmographic (PPG) pulse oximetry, providing a convenient and accessible alternative.

Thus, pulse oximeters are non-invasive devices that measure the percentage of oxygen carried by the hemoglobin in arterial blood rather than the concentration of oxygen dissolved in the blood [53].

The system can be based on transmission or reflection (transmission or reflection oximetry). The former, wherein light traverses a finger or earlobe to reach the detector, is predominantly employed in clinical settings. The latter, involving the reflection of light by the skin, finds more extensive use in remote monitoring, offering enhanced wearability as well.

The fundamental principle underlying this technique is based on the distinct physical properties of oxygenated and deoxygenated hemoglobin and the resulting variations in absorption spectra. Deoxygenated hemoglobin absorbs more light in the range of 600–750 nm, whereas oxygenated hemoglobin absorbs more between 850 nm and 1000 nm [53]. Thus, the device is composed of two light sources in these two ranges, along with a photodetector for measuring light absorption. Making use of the Lambert–Beer law, the oxygen saturation can be defined as the ratio of the concentration of oxygenated hemoglobin to the total concentration of hemoglobin in the blood.

**Figure 1:**
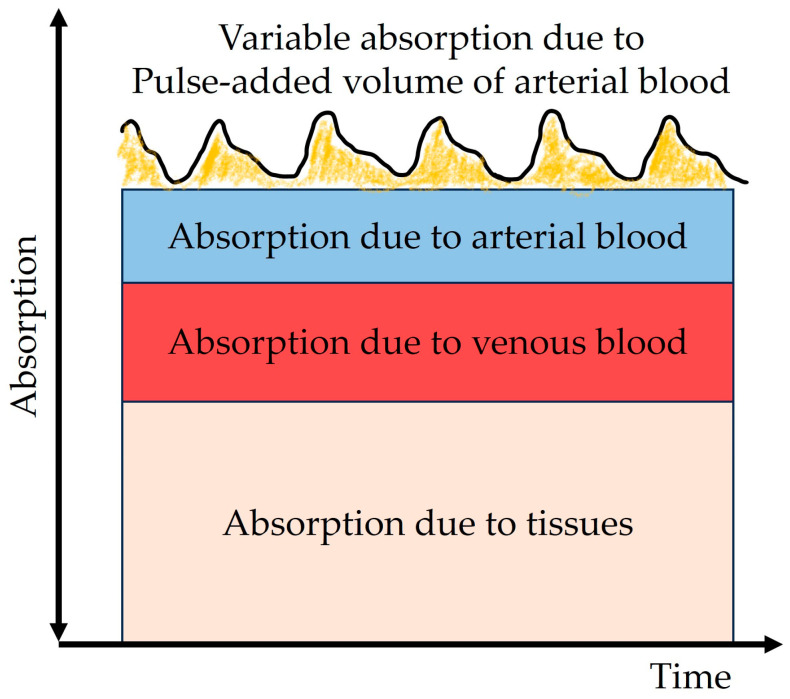
Representation of the non-pulsatile and pulsatile blood components determining the plethysmography pulse range measurement.

Pulse oximeters can be used to measure the arterial pulse rate as well [53]. Such a measurement is based on the identification of absorbance peaks over time and is representative of the pulsatile arterial component only, since the absorptions of other blood components (venous blood and arterial blood) and tissue are constant (Figure 1).

Pulse oximeters are renowned for their ease of use [8,36] and their low cost. They are the most clinically used devices for non-invasively assessing oxygen in the human body and can be integrated in body sensor networks together with other sensors [54].

However, there are factors affecting these readings, such as motion artifacts [10] or the color of the epidermis. There is also a possibility of a shift in the dissociation curve based on the calibration model applied, and a consequent erroneous interpretation of PO_2_ with respect to SpO_2_ [10] or reduced accuracy in the extremes of the dissociation curve, even though these are the points of potential clinical relevance [1,4].

### 3.4. Transcutaneous Monitoring

**Figure 2:**
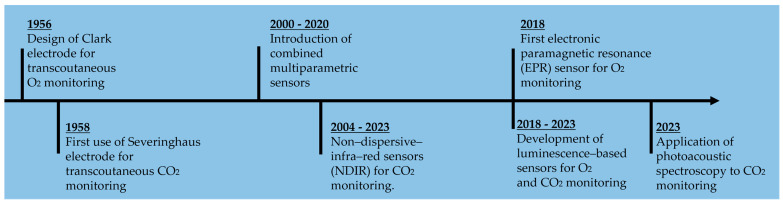
Timeline of oxygen and carbon dioxide sensors for transcutaneous gas monitoring.

Since the 1970s, the adoption of mechanical ventilation in the Neonatal Intensive Care Unit (NICU) has expanded, revealing rapid fluctuations in arterial blood gases that cannot be adequately captured through intermittent arterial blood draws. This clinical imperative has driven advancements in sensor technologies specifically tailored for transcutaneous monitoring. In Figure 2, a timeline illustrating the key milestones in the development of transcutaneous oxygen and carbon dioxide sensors is presented.

Traditional technology for transcutaneous monitoring is based on electrochemical sensors, described in detail in Section 4.1 and Section 5.1. They require re-membraning [39], large and expensive equipment, lengthy and frequent bedside calibration procedures [40], and are suitable mainly for immobile patients [55]. Moreover, they are susceptible to drift [40], requiring training to distinguish drift from other factors related to the patient [10].

To overcome the electrochemical working principle’s limitations, starting from the 2000s, research focused on the implementation of alternative technologies [8,56]. In Table 1, all the investigated sensors are elucidated in terms of their operational principles, associated advantages and disadvantages, and potential future directions.

In general, the advantages of transcutaneous monitoring technologies can be summarized in the following points:

- Non-invasive
- Continuous
- Fast readings
- Early recognition of poor tissue perfusion
- Early recognition of respiratory complications
- Estimation of systemic perfusion
- Decreased risk of operator error
- Not influenced by ventilation–perfusion disorders
- Possible combination for multiparametric sensors (for example pulse oximetry).

An additional stride in advancing this framework entails interest in wearable devices for conducting transcutaneous measurements.

Wearable sensors, first thought to track physical activity and well-being [1], are also gaining considerable use in personal healthcare monitoring applications [57]. They can particularly lead to a great advance in blood gas monitoring, due to the perspective of changing from a bulky bedside device to a miniaturized wearable device [3], to reduce the strain on medical resources [18], to improve the outcomes of treatment, and, most of all, to derive frequent alterations in respiratory parameters [1], such as in COVID-19, sleep apnea, and COPD [3,6]. Therefore, the introduction of non-invasive [34,57], low-cost, and wearable devices with miniaturized sensors [55,57] is a desirable perspective for transcutaneous sensing, opening up the possibility for disease self-management.

**Table 1:** Non-invasive past and recent technologies for oxygen and carbon dioxide transcutaneous monitoring, highlighting each technology’s working principle, technical specifications of representative examples (if available), advantages, disadvantages, eventual future developments, and target gas.

Working Principle	Device	Technical Specification (Examples)	Advantages	Disadvantages	Future Development	Target Gas
Electrochemical	Clark’s electrode	Drift: 1–2 mmHg/h [30] Response time to 99%: 40 s (with polyethylene membrane, ref. [58])	Accuracy	Heating Oxygen consumption Calibration	-	O_2_
	Severinghaus electrode	Sensitivity: linear (range 1.38–11.37% CO_2_) [58] Response time: 2 min after a rise in CO_2_, 4 min after a fall in CO2 [58]	Accuracy	Heating Oxygen consumption Calibration	-	CO_2_
	ISFET [34,59]	Drift < 0.23 mV/h [59] Response time: <1 min [59], 60 s [34] Sensitivity dependence on temperature: 0.26 ± 0.1 mV/log(PCO_2_)°C [59]	Miniaturized	Drift of the reference electrode Temperature dependency Few hours or days of use	-	CO_2_
Luminescence-based	Bandage-like sensor (2019) [60]	Response time: 1.5 faster respect to a commercially available tcpO_2_ sensor Sensitivity (=I_0_/I_30_): 1.61 Data fitting correlation coefficient (R^2^) = 0.9951	Fast response Accurate Sensitive Wearable Flexible	Heating One hour of use	Increase time of use	O_2_
	Integrated readout circuit [18]	Measurement range: 0–150 mmHg of PO_2_ Power consumption (LED driver): 20 mW	No heating Low power consumption	Requires a stronger LED driver than PPG sensors	-	O_2_
	Intensity- and lifetime-based sensor [61,62,63]	Measurement range: 0–160 mmHg of PO_2_ [64] Response time: 15 µs in room air (PO_2_ = 160 mmHg) Calibration time: 20/30 min [63]	No heating Real-time Insensitive to motion artifacts	Temperature-dependent Long calibration	-	O_2_
	Fluorescent thin-film-based [3]	Measurement range: 0–75 mmHg PCO_2_ Power consumption of the circuit board: 64.33 mW Response time (depending on the PCO_2_ level): 6–20 min	Miniaturized No heating	Need a saline solution for stability	-	CO_2_
	Dual lifetime referencing [65]	Measurement range: 0–75 mmHg CO_2_ Robustness against excitation length variation: luminescent ratio ∆% ∼1.6% across the PCO_2_ range Power: 541.25 mW	No heating No common noise	After 76 mmHg f-LDR becomes useless	Use a square wave for f-DLR technique Methods to compensate for the errors	CO_2_
	Wearable prototype device [55]	Photostability: 120 min (under air conditions) Measurement range: 0–50 mmHg CO_2_ Model fitting: R^2^: 0.9808 Sensitivity: 0.13/mmHg	Reduced volume and thickness Photostable Biocompatible	Dependency on temperature	-	CO_2_
Electronic Paramagnetic Resonance (EPR)	SPOT chip [7]	Sensitivity (mG/mmHg): 16.0–0.161 × T(C) Precision: SD = 5.5 mmHg Measurement range: 0–160 mmHg PO_2_ Reproducibility: 1 year	Robust No heating Highly sensitive Reproducible measurements	Validated on a small number of volunteers Temperature-dependent	More in vivo validations	O_2_
Non-Dispersive Infra-Red (NDIR)	Photoreaction chamber with pyroelectric sensor [66]	Response time: <2 s Sensitivity: 4.3 mV/mm Measurement range: 1000–20,000 ppm of CO_2_	Sensitive even at reduced dimensions Fast response time (<2 s)	Only in vitro Not developed yet to be applied on human skin	Include a heating wire to collect CO_2_ from the skin Include a vacuum pump	CO_2_
	Wearable CO_2_ monitor [40]	(Cozir^®^ NDIR CO_2_ [67]) Response time: 30 s Accuracy: ±70 ppm Dynamic range: 0–5% Lifetime > 15 years	No heating Wearable Accurate Good longevity Low power consumption	-	Self-calibration Diffusion optimization Signal processing Algorithms	CO_2_
	Prototype for a miniaturized monitor [43]	Measurement range: 0–120 mmHg PCO_2_ Response time: 4 s Noise floor: 30 mV_RMS_	No heating Precision Response time 4 s	Only in vitro	-	CO_2_
	Rate-based monitors [4,15,27]	O_2_ resolution: 1-ppm [27]; CO_2_ resolution: 2-ppm [27] S/N O_2_: 1.5 Δppm [27]; S/N CO_2_: 3 Δppm [27]	No heating Short response time (<2 min)	Bulky and high power requirements to be wearable Calibration	Test on adults	CO_2_
	Innovative design for a wristband wireless device [68]	Power autonomy: 6 h Correlation coefficient (VS Sentec, Essex, UK): 0.47 Correlation coefficient (VS Lifesense Nonin, Plymouth, MA, USA): 0.32	Overestimation of CO_2_ Wearable Autonomous Correction of humidity and temperature	Heating Calibration	-	CO_2_
	CAPNO device [69]	-	Correction of humidity temperature	Heating Calibration	-	CO_2_
Photoacoustic spectroscopy	Photoacoustic [70]	SNR: 254.04 Minimum detection limit: 2.6 ppmv (at integration time 365 s) Sensitivity: 636.9 ppmv/V	Long-term stability Sensitivity Selectivity No heating	Temperature influences the f_0_ of the QTF	-	CO_2_

Another interesting possibility for wearables is their customization following the individual’s characteristics to reach a high accuracy, exploiting machine learning techniques coupled with personal calibration [1].

### 3.5. Implantable Sensors

Although the object of this review concerns non-invasive transcutaneous sensors, it is worth mentioning implantable sensor technology. This is a very innovative and recent frontier in the field of gas monitoring.

The first implanted sensor tested in vivo was a device based on an electrochemical sensor published in 2020 by Jamie R.K. Marland et al. [71]. It was designed by silicon-based fabrication techniques to allow for miniaturization, reproducibility, and low costs. Its surface was coated with a thin film of Nafion to prevent biofouling and allow for the transport of water and protons, thus supporting the electrochemical reactions. It was based on a three-electrode cell: the platinum working electrode, where the oxygen reaction occurred, the Ag/AgCl reference electrode, and a platinum counter electrode. The sensor provided a linear response to oxygen, although some sensitivity to biofouling and urate was present, and a lifespan constrained to a range spanning from hours to days. The aim of this technology is to provide continuous measurements of hypoxic regions in tumors to correctly ‘paint’ the dose during radiotherapy treatments. Indeed, hypoxia occurs when there is a disorganized growth of blood vessels, such as in the case of tumors, and causes an increase in the resistance of cancer cells to radiation [71,72]. Since positron emission tomography (PET) and magnetic resonance imaging have limited spatial resolutions, implantable miniature sensors are being developed to be implanted directly into tumors and provide a real-time hypoxia map to correctly deliver the dose to patients [71,72]. This approach can be used also in many other pathological cases such as non-healing wounds or hypovolemic, cardiogenic, and septic shock [71].

Another implantable sensor was presented the same year by Soner Sonmezoglu and Michel M. Maharbiz, based on luminescence [73]. This system could sense O_2_ in deep tissue and, thanks to the use of a single ultrasound link to power and communication with the device, it had a very small size of 4.5 mm^3^ that guarantees its potential for chronic use and minimal tissue damage. The measurement system consisted of a μLED for optical excitation, a biocompatible film for the encapsulation of O_2_-sensitive luminescent ruthenium (Ru) dyes, an optical filter, and a miniaturized IC. During its functioning, the shift in phase between emission and excitation light, a function of luminescence lifetime (described later in detail), was related to the O_2_ concentration. Its validation and measurements were accomplished in vitro and, according to the authors, it had the lowest power consumption and the smallest volume of any other system presented at that time.

Despite these examples, there is still a considerable journey ahead to introduce these types of sensors into clinical practice, requiring extensive further research.

## 4. Transcutaneous Oxygen-Sensing Technologies

In the following subsections, different sensor technologies for oxygen detection are described.

### 4.1. Electrochemical Sensors

The Clark’s electrode was introduced to measure dissolved oxygen in 1956, almost seventy years ago, and it is still the basis of modern clinically used transcutaneous devices [74].

**Figure 3:**
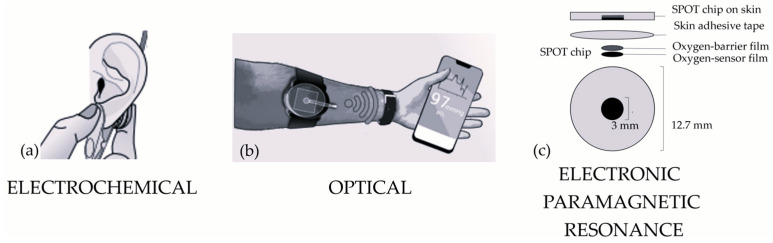
Transcutaneous sensors for oxygen detection. (**a**) Electrochemical sensor for transcutaneous oxygen detection positioned at ear lobe; (**b**) optical thin film sensor (adapted from [64]); and (**c**) SPOT chip, exploiting electronic paramagnetic resonance (adapted from [7]).

The sensor consists of a platinum cathode and silver/silver chloride anode, suspended in a potassium chloride solution within a cellophane compartment. An oxygen-permeable membrane separates the electrolyte solution from the sample to be measured. A voltage is applied between the electrodes and a redox reaction takes place. At the anode, chloride ions from the electrolyte react with silver, generating silver chloride and free electrons. These electrons participate in the reduction in oxygen on the negatively biased (usually −0.8 V) platinum cathode, producing water. The electrons’ flow between the electrodes generates a current, and its magnitude is linearly proportional to the partial pressure of oxygen in the sampled fluid [75]. A representative image of the assessment at the earlobe is shown in Figure 3a. It provides accurate measurements [58] without the need for an arterial blood draw and permits continuous monitoring.

However, as previously mentioned, it has different drawbacks. First, the temperature must be constant [58] and reach 43–44 °C [10,30,36], causing risks of burn wounds and pressure-induced necrosis, especially in neonates [36]. Then, it suffers from measurement drift, due, for instance, to electrolyte consumption, thus needing frequent membrane changes and calibration, reducing its overall usability [10]. An increased deviation of pressure measurements was observed in the presence of local tissue fluctuation, particularly for small local tissue blood flow [31]. Of note, another remarkable limitation of the Clark electrode is the consumption of oxygen required to perform the measurement [7,10], leading to an underestimation of PaO_2_ in stagnant samples. Controlling the sample renewal rate or flow rate can influence the accuracy of the measurements. Increasing the thickness of the membrane can moderate this effect, even if penalizing the response time.

Against this historical amperometric sensor, new emerging technologies show great potential for transcutaneously assessing O_2_ tension.

### 4.2. Optical Sensors

Recently, luminescence-based technologies have been emerging for transcutaneous monitoring due to their useful advantages with respect to classic electrochemical sensing.

In O_2_ monitoring, these sensors have undergone rapid growth and are in the process of replacing the Clark electrode in many fields. This transition is also facilitated by their ability to work in hostile environments and chemical conditions [76].

The foundational principle of this approach lies in luminescence quenching. Luminescence quenching consists of the emission of light from a luminescent substance, such as a fluorophore or phosphor, reduced or “quenched” by the presence of a quencher molecule, for instance, O_2_ [37].

When the luminescent material is hit by UV or visible light, electrons absorb photons and go into an excited state. This state is unstable, so electrons tend to go back to the ground state, emitting excess energy in the form of photons of light. If the luminescent dye is made of fluorophores, the phenomenon is called fluorescence, whereas, if it is made of phosphors, this is phosphorescence. Fluorescence is a very rapid type of luminescence that occurs within 10^−8^ s, while phosphorescence has a greater decay time [77,78]. When luminescent molecules encounter a quencher, they collide, and the quencher absorbs some of the excess energy from the luminescence dye, causing a quicker return of the electrons to the ground state. This results in a decrease in the intensity and duration of the emitted light. This reduction is directly proportional to the concentration of the quencher, and it is governed by the Stern–Volmer equation [56,61,76,79]:

(1) $$\frac{I_{0}}{I}=\frac{\tau_{0}}{\tau }=1+K_{SV}C_{O_{2}}\;$$

where *I*_0_ and *τ*_0_ are the reference values of the luminescence intensity and decay time of the probe in the absence of O_2_, *I* and *τ* represent the respective values in the presence of O_2_, *K_SV_* is the Stern–Volmer constant, and *C_O_*_2_ is the concentration of O_2_ in the sample.

There are several advantages of luminescence-quenching techniques. They are reversible, since they do not alter the optical probe, oxygen is not consumed, and sensors can be designed to detect wide ranges of concentrations or partial pressures [56].

In the realm of luminescence quenching, two kinds of measurements can be performed, one based on intensity and the other on lifetime measurements. In intensity-based detectors, intensity can be altered by different confounding factors, resulting in the output signal variability. Such factors are the sensor geometry [61], the detector’s photosensitivity [65], the excitation intensity, and the material’s photobleaching [61,65]. On the contrary, lifetime measurements, exploited from the 1990s [80,81], grant more accurate results and are insensitive to these factors [65]. Lifetime-based approaches can be exploited through either a time domain method, measuring the decay time of the emitted light, or a frequency domain method, measuring the phase shift between the excitation and emission light. The same principles apply to both O_2_ and CO_2_ measurements [65].

Extensive recent research efforts have been focused towards developing materials with highly tunable structural and spectral properties, rendering them well-suited for various applications in biological environments and wearable technologies.

In 2018, a new phosphorescent quenching sensor was presented [56], and, to the best of the author’s knowledge, this was the first work on a wearable and flexible PtO_2_-monitoring system based on a luminescent gas-sensing mechanism. The sensor was composed of a sensing film, an organic light-emitting diode (OLED) as a light source, a cellophane paper used as a filter to reduce the optical noise, and an organic photodiode (OPD) as a light detector. The sensing film was composed of a polymer matrix in polystyrene (PS), in which the sensing dye, platinum porphyrin, doped with TiO_2_, was embedded. In fact, TiO_2_ increased the sensitivity thanks to its light-scattering capability. This sensor was validated against a traditional gas monitor (Periflux 5000, with a PF5040 unit, PERIMED Co., Ltd., Stockholm, Sweden [82]) and proved to be more comfortable and less sensitive to body movements. Furthermore, it has a high applicability on any part of the body, without restrictions in terms of area curvature, for example, the thumb. Nevertheless, the sensor is able to measure O_2_ approximately from 25 to 35 mmHg, which is not physiological.

Thus, in 2019, the same authors proposed a new, flexible, bandage-like, photoluminescence (PL)-based transcutaneous oxygen sensor [56,60], with the addition of a film heater to increase the skin temperature and reach a physiological range of measurement. A further addition to the previous version of the sensor consisted of a polyvinylidene chloride (PVDC) film as an encapsulation layer to improve the sensitivity of the photoluminescent O_2_-sensing film by minimizing the PL quenching effects of ambient atmospheric O_2_ [60]. A micro-LED array embedded into a polydimethylsiloxane (PDMS) film was introduced, replacing the OLED, to achieve stable light emission. This permitted achieving faster and more accurate PtO_2_ measurements over several cycles for as long as 60 min [60], which was not possible with the previous device. Therefore, as suggested by the authors, future improvements may regard the minimization or, eventually, the eradication of the ambient-air-induced degradation of the OPD through the development of transparent flexible encapsulation films for the sensor to be able to operate beyond 60 min and be truly wearable over a long period of time [60].

In 2020, the first readout IC (integrated circuit) for measuring PtO_2_ based on fluorescence lifetime measurements was presented [18]. The system was composed of an LED driver that activated the blue LED with a peak wavelength of 450 nm and a Pt-porphyrin film which emitted red light at a peak wavelength of 650 nm, whose intensity and lifetime were inversely proportional to the concentration of O_2_ around the film. The IC structure included a transimpedance amplifier (TIA), designed to convert the current generated by the photodiode (PD) into voltage, and a variable gain amplifier (VGA) [6]. The whole readout occupied 1.04 mm^2^ and its performance was evaluated both in vitro and in vivo by placing a fingertip on the sensor, and it was found to successfully measure the partial pressure of O_2_ in the range of 0–150 mmHg, which optimally covers the human partial pressure of oxygen range between 75 and 100 mmHg.

The negative aspect is that, since the intensity of the emitted light from the film is lower than the light received from the photoplethysmographic (PPG) sensors, it requires a stronger LED driver, which consumes more power. Considering its nature as a transcutaneous sensor, it nevertheless maintains a low level of power consumption.

In the same year, Cascales et al. proposed a wireless wearable transcutaneous oxygen monitor based on intensity and lifetime measurements, combining their advantageous aspects. It consisted of a small device that weighed less than 30 g, as shown in Figure 3b [61,62,63]. The excitation light was given by two UVA LEDs, and an oxygen-sensing film with different layers was used. It was composed of a semipermeable and transparent membrane, which partially isolated the skin from atmospheric oxygen, a polypropyl methacrylate (PPMA) film with embedded metallic portions and oxygen-sensing dye, where the true oxygen sensing happened, and a layer to collect emissions and optically insulate the sensor from exogenous light sources. Emitted light was detected using a PIN photodiode and the Stern–Volmer equation was used with a coefficient K_eff_, which is temperature-dependent:

(2) $$X=\frac{X_{0}}{{1+K}_{eff}\cdot PO_{2}}+X_{OFF}$$

where X is the measured light (either *I* and *τ*), X_0_ is the value of X in the absence of oxygen, X_OFF_ is a non-oxygen dependent offset, K_eff_ is the oxygen diffusion coefficient of the multilayer film, which has a quadratic temperature dependency, and PO_2_ is the oxygen pressure.

Two measurements of oxygen pressure can be obtained, one from intensity and the other from lifetime. The estimate related to lifetime is more robust against motion, film positioning, and photobleaching, but has a lower signal to noise ratio (SNR). Instead, the estimate related to intensity is more sensitive to the same phenomena, but has a higher SNR.

The last version of the sensor allows for measuring PtO_2_ in almost real time [63].

### 4.3. Electronic Paramagnetic Resonance Sensors

A novel technology for the measurement of transcutaneous O_2_ was presented in 2018, based on the principle of electronic paramagnetic resonance (EPR) [7]. Electron paramagnetic resonance (EPR) oximetry exploits the paramagnetic properties of molecular oxygen arising from the two unpaired electrons it has in its ground state. EPR oximetry involves the use of a paramagnetic EPR spin probe. This spin probe is a substance with unpaired electrons, and it interacts with the unpaired electrons of molecular oxygen through spin exchange. The spin exchange interaction is sensitive to the concentration of oxygen in the local environment. Specifically, the relaxation rate of the spin probe, the time needed to return to a lower-energy state, increases as a function of the oxygen partial pressure (PO_2_). This leads to an increased spin–spin relaxation rate, the time needed for the coherence among the spins of the paramagnetic electrons to decay. The increased spin–spin relaxation rate results in line broadening of the EPR resonance line observed in the EPR spectrum. The width of the EPR resonance line correlates with the oxygen tension in the local environment, making the width of the signal the physical quantity to assess. In the study, an oxygen-sensing skin adhesive film called SPOT chip (superficial perfusion oxygen tension) was used, and is represented in Figure 3c. It had a 3 mm diameter and 60 μm thick circular film composed of a stable paramagnetic oxygen sensor, with an oxygen barrier on one side to insulate the chip from oxygen in ambient air. It quantified PtO_2_ through the linewidth of the EPR spectrum, with which it was linked through a linear relationship in the range of 0–160 mmHg, without heating. Moreover, the sensitivity of the sensor decreased with temperature, and the authors provided an experimentally obtained formula to account for this dependency.

The sensor resulted in giving robust PtO_2_ measurements and was highly sensitive to low oxygen levels and reproducible for long term monitoring. However, this sensor needs in vivo validation on numerous samples to be considered as a reliable technology and potential substitute as an electrochemical sensor for transcutaneous monitoring, even if it is still unsuitable for a wearable device.

## 5. Transcutaneous Carbon-Dioxide-Sensing Techniques

In the following subsections, sensors for the transcutaneous monitoring of CO_2_ through electrochemical and optical technologies are illustrated.

### 5.1. Electrochemical Sensors

#### 5.1.1. Severinghaus-Type Electrode

Severinghaus developed a technique known as transcutaneous monitoring, allowing for the continuous and non-invasive monitoring of CO_2_ levels, modifying the Stow electrode, which was composed of a rubber membrane on a glass electrode with water in between [58,81]. In fact, Severinghaus proposed a Teflon membrane in combination with a cellophane layer, soaked with sodium bicarbonate (NaHCO_3_) and sodium chloride (NaCl), which increases the stability of the silver reference electrode and improves its conductivity. This cellophane layer serves the purpose of retaining a more substantial water film between the glass and the Teflon. This system exhibits a superior stability, approximately twice the sensitivity, quicker response times, and significantly reduced drift.

The Stow–Severinghaus technique is the current state-of-the-art measurement method for continuous PtCO_2_ [34], giving an accurate estimation of carbon dioxide levels [17,58]. It is based on the linear relationship between pH and the logarithm of PCO_2_ [17,32,83]. When hemodynamic conditions remain stable, the outcome of this technique provides a measurement that corresponds to the partial pressure of CO_2_ in the cutaneous capillaries, and studies have demonstrated a strong correlation between them within the range from 32 to 66 mmHg [84].

However, it is important to acknowledge the limitations of the Stow–Severinghaus technique. Improper electrode placement, the presence of entrapped air bubbles, equipment errors, or calibration issues can introduce inaccuracies into its measurements [1].

Frequent calibrations are needed both after prolonged monitoring and when the sensor is repositioned [14,34]. This technique also suffers from drifts over time [31,34,40] and requires membrane replacement [39,40], as already stated before. Furthermore, the Severinghaus electrode requires heating, typically at 42 °C, with all the drawbacks listed in Section 2.1. Furthermore, CO_2_ blood changes need about 2 min [85] to be reflected by PtCO_2_. This could be a limitation in this kind of monitoring for surgery patients [14].

Finally, Stow–Severinghaus-type electrodes may show discordance between the measured PtCO_2_ and PaCO_2_ in highly dynamic situations, therefore undermining the reliability of the measurement during, e.g., physical exercise, a problem that was accounted for by enabling a multiple regression model to develop a correction factor for PtCO_2_ [23].

In recent years, an increasing number of researchers have shifted their focus towards exploring alternative approaches to circumvent the limitations associated with the traditional Stow–Severinghaus electrode.

#### 5.1.2. Ion Selective Field Effect Transistor (ISFET)

Remaining in the context of electrochemical sensors, a possible alternative to the Stow–Severinghaus electrode is represented by ISFETs. ISFETs, or Ion-Sensitive Field-Effect Transistors, represent a type of MOSFET (Metal–Oxide–Semiconductor Field-Effect Transistor) in which the gate is responsive to carbon dioxide, CO_2_, which diffuses from the analyte through a PTFE membrane to the upper layer of the gate. The hydration in the electrolyte solution generates Hydronium ions (H^+^), consequently influencing the solution’s pH. By incorporating a reference electrode, it becomes possible to measure and apply the grid-source potential, thereby creating a functional pH meter.

Although these sensors show the advantage of being miniaturizable, they are subject to the same drawbacks as the Stow–Severinghaus electrode, the drift of the reference electrode, and temperature dependency [34]. Thus, conductometric and ISFET-based sensors, as well as the Stow–Severinghaus electrode, are not usable for more than a few days. Consequently, certain limitations in electrochemical sensing methodologies open up avenues for alternative technologies relying on distinct underlying principles.

### 5.2. Optical Sensors

#### 5.2.1. Luminescence-Based Sensors

Among recent technologies for the measurement of transcutaneous gases, optical sensors based on luminescence assume an important role [77,78].

In 2021, Tufan and Guler presented a thin fluorescent film-based miniaturized transcutaneous carbon dioxide sensor [3], based on an LED with dominant wavelength of 470 nm and on a photodiode for detecting the green light emitted by the gas sample with a wavelength of 520 nm. The device comprised a sensing film that made heating unnecessary, whereas it required storage in saline solution (pH = 5.5) for chemical stability throughout the measurement. A PDMS membrane, which is impermeable to liquids and permeable to CO_2_, was added to the device. The fluorescence intensity detected by the photodiode was inversely proportional to the PtCO_2_ to which the film was exposed for the quenching effect. This design, which was validated in vitro, has the widest PCO_2_ measurement range when compared to other sensors in the literature.

As already stated before, intensity measurements of fluorescence can be altered by confounding factors, while lifetime measurements result in giving more accurate signals and insensitivity to these factors [61,65]. However, the lifetime of fluorophores happens in nanoseconds, requiring special optical instrumentation and high-speed electronics, not feasible for low-power and miniaturized devices [65].

To account for this problem, Tufan and Guler, in 2022, proposed another luminescent-based PtCO_2_ sensor [65], this time based on dual lifetime referencing (DLR).

**Figure 4:**
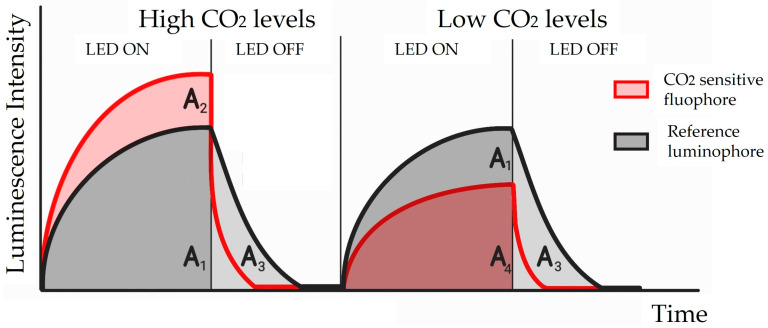
Time domain representation of dual lifetime referencing signal for different values of carbon dioxide. A_1_ is the signal obtained from CO_2_-insensitive luminophore, while A_2_ and A_4_ are the signals of the fluorophore. A_3_ represents the total luminescence during the period in which the LED is off (Adapted from [65]).

In addition to a fluorophore, the sensor incorporates a secondary luminophore called the reference, in Figure 4, insensitive to the analyte, which has a significantly longer lifetime, typically in a µs regime, than that of an analyte-sensitive fluorophore. Since the two luminophores are equally affected by the confounding factors, they could be cancelled out by taking the ratio of the total luminescence of both the CO_2_-sensitive fluorophore and the reference luminophore. In the readout circuit, a phase detector based on a digital phase discriminator is used to find the phase shift in the luminescence and the duty cycle of its output pulse train is proportional to the phase difference.

The authors explored two different techniques, the time domain DLR (t-DLR) and the frequency domain DLR (f-DLR), and managed to successfully quantify PCO_2_ in a range from 0 to 76 mmHg based on the t-DLR technique, whose signal is schematically reported in Figure 4. If a square wave was chosen instead of a sinusoidal wave as the excitation signal, an error in the phase measurement was introduced because of the harmonics present in the square wave. So, when CO_2_ increased, f-DLR became useless. Nevertheless, the possibility of using square wave excitation for the f-DLR technique and methods to compensate for the errors is a topic of future investigation.

The same first author, Tufan et al., presented, in 2023, a wearable device for PtCO_2_ monitoring based on the t-DLR sensing methodology. After gas cell experiments confirming its accuracy in the detection of CO_2_ partial pressure changes in the clinically significant range, it was tested on the forearm of one volunteer during a hyperventilation maneuver. The device was perfectly able to detect changes in the PCO_2_ when transitioning the sensor from ambient air to the skin, despite the requirement for a 30 min stabilization period. However, it failed to register changes in PtCO_2_ caused by hyperventilation. This could be attributed to a slow diffusion rate influenced by several factors, primarily the absence of a heating mechanism and the location of measurement. Positioning the sensor on the fingertip allowed for the detection of PtCO_2_ variations induced by hyperventilation [86].

Among luminescence-based sensors, dye-based sensors are worth mentioning. They rely on CO_2_’s influence on the pH of aqueous solutions, which will be translated in the change of the optical properties of a pH-sensitive dye [34,87]. Furthermore, the chosen dye is protonated, and anionic forms have different absorbance or luminescence spectra; thus, different wavelengths can be used for their measurements. In terms of characteristics, dye-based sensors’ response time is below 1 min, their accuracy below 1%, and they cover the full range of CO_2_ [34].

Dye-based sensors can be categorized as dry and wet sensors. The difference is that the key part is a liquid, usually aqueous, layer or a solid-state system for wet and dry, respectively [88]. In dry sensors, the dye is usually incorporated into a polymer matrix, which is then cast on a solid support [34,87]. Dry sensors produce results far better than those of wet ones, in terms of response time and long-term stability.

Another work on a wearable transcutaneous CO_2_ sensor enhancing fluorescence quenching was published in 2022 by Cascales et al. [55]. The device was based on a fluorescent pH indicator (8-hydroxy-1,3,6-pyrenetrisulfonic acid trisodium salt or HPTS) embedded into hydrophobic polymer matrices. The layers were a breathable and white silicone film as an optical insulator, preventing external lighting from affecting the measurement, a PPMA-based sensing film, and a transparent semipermeable film, used to impede the room’s air from interfering with the transcutaneous measurements. This configuration presented a reduced volume and thickness and, hence, fast equilibration to the CO_2_ concentration of the skin. In addition, the film resulted in being photostable and insensitive to humidity. The film was excited with two high-intensity LEDs at 405 nm and 470 nm and its emissions were sampled via a PIN photodiode. In the presence of CO_2_, the dye anion was converted into its protonated form, which did not show fluorescence when excited at 470 nm, whereas the dye exhibited maximum excitation at about 400 nm. The emission from the films exhibited CO_2_-dependent characteristics when excited at a wavelength of 470 nm. An analysis of the excitation spectra revealed an isosbestic point at approximately 405 nm. Consequently, this wavelength was selected as a reference to employ a ratiometric approach, ensuring a measurement unaffected by photobleaching effects and resistant to motion artifacts. In the physiological range of carbon dioxide, the sensing film’s fluorescence was shown to be highly sensitive to CO_2_ pressure changes, even if a conversion from relative values into the mmHg scale was not performed.

Despite these advantages, this technique still shows a dependency on temperature. In fact, the breathability of the white film was greatly enhanced by increasing the temperature to above 40 °C.

#### 5.2.2. Non-Dispersive Infrared Sensors

Non-dispersive infrared (NDIR) sensors are the most used for CO_2_ monitoring [10], and, according to Vafaei et al., the NDIR-based CO_2_ gas sensor is the most appropriate and preferable sensing mechanism for detecting and monitoring CO_2_ gaseous emissions [89], used to avoid the drawbacks of traditional transcutaneous CO_2_ sensors.

The principle of non-dispersive infrared measurement is the Lambert–Beer law, according to which, the infrared radiation propagating in a gas medium is attenuated at certain wavelengths due to the absorption of the gas.

Studying the intensity of the transmitted radiation with respect to the intensity of the incident one, the absorbance coefficient of the material is computed, and it is proportional to the path length and the concentration of the gas in the sample [90]. Therefore, a high concentration implies an increase in the optical density of the material and a decrease in the intensity of the transmitted light.

Gaseous carbon dioxide presents an absorbance peak at 4.26 μm, a wavelength of absorption different from that of other common gases. Because of that, NDIR sensors are very specific and able to reach low levels of detection [34,43].

An NDIR transcutaneous measuring system was proposed in 2004 by Hwan-Joo et al. [66], composed of an IR lamp, an optical filter, an optical reaction chamber, a pyroelectric sensor, and a signal processing chain. The purpose of the authors was to assess whether the volume of the equipment could be reduced, making it suitable to be applied on the skin and portable, without affecting its sensitivity. In particular, they assessed the length of the photoreaction chamber, decreasing it down to 1 mm, and the results proved satisfactory, as the absorbance could be measured in a wide CO_2_ concentration range. The absorbance was proportional to the concentration of the gas, and to find the pressure, a second step was needed, exploiting the Henry’s law, even if this procedure was not detailed in the article. In fact, concentration and partial pressure are related to each other by a proportionality factor, the Henry’s constant, specific for the gas or liquid under analysis and dependent on temperature [66].

Water vapor and ambient temperature interfere with the output of an NDIR sensor. To improve their performance, especially in the case of wearables, two different solutions were proposed by Pierre Grangeat et al. in 2019–2020.

The first one, named CAPNO, is focused on a computational [68] method. In this device, the contribution of the ambient temperature is removed by computing the measurement of the thermopile conducted when the IR source is switched off, while the humidity disturbance on the measurement is compensated through a dual wavelength optical measurement. In detail, two channels are implemented: one sensitive to carbon dioxide and the other one to no gas. The latter is used as a reference. By comparing the performance of the CAPNO device with two reference devices (an electrochemical electrode and an end-tidal CO_2_ sensor), a strong intercorrelation was found for some of the samples, even though the longer response time and the influence of ambient air represent shortcomings.

The second solution proposed regards an innovative design for a wristband wireless device [69]. Firstly, a differential measurement was performed, choosing 4.26 µm and 3.91 µm wavelengths, corresponding to the absorbance peak of the gaseous carbon dioxide and to a minimum of the absorption intensity for water, respectively. Secondly, a thermo-fluidic channel, included in the structure of the sensor, functioned to replace the diffusion of the carbon dioxide gas with convection. The convection principle was made possible thanks to the presence of a collection cell in contact with the outside air, which collected the outcoming gas from the heated skin. The two remaining components were a measurement cell composed of two thermosensitive sensors and a light source, and a gas outlet cell that allowed the gas to be released. Finally, a microcontroller collected the outputs of the temperature, pressure, and humidity sensors and corrected any variations in the signal processing, making the wearable device autonomous. Once again, the experimental device was validated by a comparison with an electrochemical and an end-tidal CO_2_ device, demonstrating a reduction in variability and robustness with respect to the workload modulation.

Other works regarding NDIR CO_2_ monitoring were presented in 2021 and 2022 [40,50,91].

One of these studies focused on the integration of a miniaturized NDIR sensor into a wristband [40], characterized by longevity, robustness, and a low power consumption. Tipparaju et al. used a Cozir^®^ NDIR CO_2_ sensor (Gas Sensing Solutions, Cumbernauld, UK), based on solid-state LED technology and characterized by a fast response time (30 s) and long lifetime (>15 years) [67]. These two features are particularly interesting if compared with a classic electrochemical sensor. The sensor also had a built-in auto-calibration system. Concerning the wristband, it was a 3D-printed plastic body that housed the sensor, which remained exposed to a miniaturized gas chamber where the CO_2_ released from the skin could diffuse. An O-ring provided air sealing to avoid gas leakage from or into the ambient air. A hydrophobic membrane was added to account for the humidity interference that occurs due to sweating. A PDMS membrane was synthesized and integrated into the NDIR CO_2_ sensor [40], which increased the sensitivity of the sensor if compared with traditional transcutaneous gas monitors using a Teflon membrane. However, the lack of a heating system will impact the performance of such a sensor.

Another solution proposed to solve the interference of water vapor was the use of an optical filter in front of the detector to select only the wavelength the gas molecules can absorb [92].

Another article of 2021 presents a new prototype of an NDIR sensor, based on a thermopile able to precisely detect variations in infrared radiations [43]. This sensor obtained good results in a pressure range between 0 and 120 mmHg, which includes humans’ typical range from 35 to 45 mmHg, in vitro. The measurement response time was 4 s. Nevertheless, the first author of this study seems to have transitioned from NDIR technology to luminescence-sensing approaches, and their contributions in this domain were detailed in the preceding paragraph.

In 2024, some of the authors of the present review proposed a wearable device for transcutaneous CO_2_ detection which encapsulates a heating system that enhances detectability and stability [93,94]. The device was tested on 30 healthy volunteers through a rebreathing maneuver of 2 min in a 2 L bag. Although additional investigation is required to tackle the obstacles associated with continuous measurements throughout a 24 h period, the device was demonstrated to be able to properly detect increases in CO_2_ induced by the maneuver, opening up new perspectives for continuous monitoring.

#### 5.2.3. Rate-Based Methods

A further comment should be reserved for the attempt to create an indirect method for retrieving PtCO_2_ based on the *rate* of increase in PtCO_2_ in a sensing device, therefore named rate-based method [4,15,27]. It has been validated both through concentration [4] and pressure [15,27] measurements, starting from the same equilibrium principle based on Fick’s diffusion law, according to which, the amount of CO_2_ that flows in the measurement system equals the amount diffusing through the skin. Since the functioning of these three sensors is the same, the following dissertation will focus only one of the three and consider only sparse features of the other two. In reference [15], Fick’s law, followed by simplifying assumptions and integration, leads to the equation:

(3) $$P=\left(\alpha p_{a}+\beta \right)t$$

where P is the measured PCO_2_, $p_{a}$ is the partial pressure underneath the skin, *t* is time, and α and β are two calibration factors, found by a two-point calibration procedure. There is a linear dependence of P, from time, and computing the derivative of Formula (3) gives:

(4) $$\frac{dP}{dt}(t=0)=\alpha p_{a}+\beta$$

the rate $\frac{dP}{dt}$ has a linear relationship with the partial pressure underneath the skin $p_{a}$. During the measurement cycle, therefore lasting no more than 1.5–2 min ([15,27], respectively) in total and possibly repeated until a stop button is pressed [27], the readings on PCO_2_ were fitted on a linear equation, from which the slope (rate) could be calculated.

In principle, this method implements an interesting solution for a wearable device, because it is based on the *initial* diffusion rate and does not require reaching a stable steady-state concentration [1,15]; therefore, the final measurement can be achieved in only 2 min (against the 15–20, refs. [4,27] for traditional transcutaneous monitors). In addition, it does not require a heating system, since the SNR of the measurement is independent from the increase in skin temperature [27]. Among the studies analyzed for the rate-based approach, a less recent sensor (2014 [4]) was tested on porcine skin, a surrogate for neonatal skin, another (2015 [15]) was tested in a neonatal ICU, and three years later (2018 [27]), an $O_{2}$ sensor was also tested in a laboratory setting on human skin.

**Figure 5:**
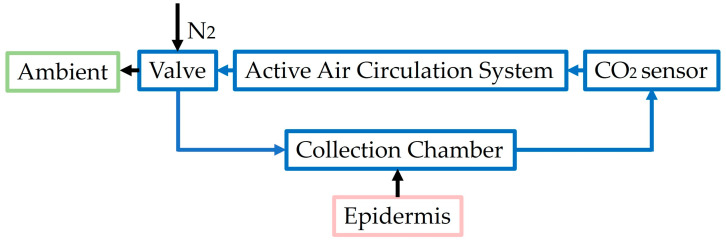
Conceptual design of a rate-based monitor, including all the components: valve, fan (or pump), sensor, and sampler chamber [1].

Nevertheless, all these devices show a similar setup, reported in Figure 5, consisting of a sampler containing the sensor (based on dual-wavelength infrared detection), a valve, which allows for switching from the $N_{2}$ flush phase (“washing” phase, lasting 30 s according to [27]) to the recirculation phase (the phase in which the measurement is performed, lasting 60 s, according to [27]), and a fan, or pump, which allows for the circulation of the gases. This setup, however, proves too bulky and power-consuming for a wearable device. In fact, only the collection chamber has dimensions suitable to be applied on the skin. Furthermore, since the calculation depends on skin diffusivity, a parameter not accurately known, the method is considered limited, with only the exception of neonates, whose skin is more homogeneous than that of adults [34]. If accurate values are required, the increase rate must be compared to ABG values, through a proper calibration, as mentioned before. If ABG samples cannot be drawn, the device can still be used as a tool to detect changes in blood gas levels and used in a wider range of population rather than only neonates [12].

#### 5.2.4. Quartz-Enhanced Photoacoustic Spectroscopy

In 2023, Yixin et al. proposed continuous real-time monitoring for CO_2_ based on quartz-enhanced photoacoustic spectroscopy (QEPAS) [70], able to obtain an electric signal proportional to the carbon dioxide concentration from the measurement of the resonance frequency of a QTF. The response time was 5 s.

QEPAS differs from photoacoustic spectroscopy as it is based on a quartz tuning fork (QTF) as an acoustic detector, which has a small size and low cost. Moreover, QEPAS sensors do not have shape and size limitations and can suppress electronic noise very well.

This sensing technique involves a laser beam to be focused by a fiber-coupled Grin collimator (FCG) to further pass through an acoustic micro-resonator (AmR) and between the prongs of the QTF, which will vibrate, producing a weak electric signal. The signal needs to be amplified by a preamplifier and demodulated by a lock-in amplifier, and finally, the second harmonic signal (2f-signal) is acquired, proportional to the concentration of CO_2_.

Due to differences between body temperature and the temperature inside the gas collection chamber, the heat conduction effect is generated, causing a variation in the chamber temperature. The temperature has a great influence on the QTF resonance frequency, and if f_0_ is changed, the output signal will also be affected. To account for this problem, the temperature inside the chamber is stabilized at a value close to the human skin, 37 ± 0.2 °C.

Further still, water vapor diffuses through the skin, determining a change in the humidity inside of the chamber, affecting, once again, f_0_. However, at the indicated temperature (37 ± 0.2 °C), humidity’s effect will be negligible on the output signal. Another precaution to avoid water vapor interference can be taken, setting the wavelength of the excitation beam at 4991.26 cm^−1^ (around 2 μm), at which CO_2_ has a water absorbance more than five times greater than that of water vapor.

Finally, body movements have been shown to exert external force on the QTF, affecting its output signal and causing some points to be irregular in the overall trend.

The sensor also needs to be calibrated after the temperature is set at the above-mentioned value. It was tested on different parts of the body and a faster CO_2_ emission rate was obtained from the left cheek skin.

Until now, results were collected from three healthy volunteers, demonstrating the device to be feasible for CO_2_ detection, even if, after 25 min of continuous monitoring, a significant change was shown compared to the signal at baseline.

## 6. Combined Transcutaneous Oxygen- and Carbon-Dioxide-Sensing Techniques

The literature reports that a combination of SpO_2_ and PtCO_2_ would be advantageous with respect to that of PtO_2_ and PtCO_2_, either as a concurrent measurement [1,36,95] or in the same sensor [8,33]. To the best of our knowledge, the combinations currently available are SpO_2_/PtCO_2,_ SpO_2_/PtO_2_/PtCO_2_, and O_2_/CO_2_ concentrations. Reference [8] showcases that combined PtO_2_/PtCO_2_ appeared in 1985, still requiring the arterialization of the cutaneous tissue. Of note, the trend in the articles analyzed to draw up this review suggests a much more recent interest in research on single sensors rather than combined ones. This could be ascribed to the fact that combined electrochemical O_2_ and CO_2_ measurements interfere with each other [10], and combined sensors are conceived mainly to replace electrochemical O_2_ measurements with optical ones.

### 6.1. SpO_2_/PtCO_2_ Sensors

In 2001, the multisensory OxiCarbo^®^ (Radiometer, Basel, Switzerland) was presented, demonstrating the earlobe as an ideal location to apply the device, both because of the accessibility of the location during intra-operative monitoring and because of the decreased arterialization time [32]. A meta-analysis conducted in 2019 [9], which was focused only on carbon dioxide, effectively demonstrated that the earlobe is the sensor location that assures the highest accordance between transcutaneous and arterial carbon partial pressure. The OxiCarbo^®^ multisensor device combines pulse oximetry, both transmissive and reflective, with transcutaneous carbon dioxide sensing through a Severinghaus-type electrode. The heating unit comprised in this last electrode, despite the general reasons already reported in Section 2.1, improves the SNR for optical measurements in non-favorable conditions, such as low perfusion and movement artifacts, and stabilizes the noise fluctuations with respect to other unheated sensors.

### 6.2. SpO_2_/PtO_2_/PtCO_2_ Sensors

Hayoz et al., in 2013, proposed a device intended for the transcutaneous determination of O_2_ and CO_2_ partial pressures [31]. To obtain better results, the device comprises a heating element that brings the skin to a constant temperature Ts, approximately around 40 °C and 44 °C. The CO_2_-sensing unit is based on the Severinghaus equation, whereas the O_2_ transcutaneous partial pressure is determined using the Clark equation.

Since both the transcutaneous CO_2_ and O_2_ partial pressure measures face substantial deviations from the effective arterial pressure, the idea was to introduce in the equations a factor F dependent on the local tissue blood flow. It is preferable to measure the flow in proximity of or beneath the transcutaneous sensor. The PtCO_2_ is computed as:

(5) $$PtCO_{2}\left(Tr,F\right)=\frac{P_{s}CO_{2}\left(Ts\right)}{10^{\left(Ts-Tr\right)xA}}-M_{s}\left(Ts,F\right)$$

where Tr is the reference temperature, usually 37 °C, and Ts is the skin temperature, M_s_ (Ts,F) is the metabolic offset as a function of both the local temperature and the correction factor, A is the anaerobic temperature factor, F is the blood flow factor, and P_s_CO_2_ (Ts) is the skin carbon dioxide partial pressure. Instead, for PtO_2_, the formula is:

(6) $$PtO_{2}\left(Tr,F\right)=Corr\left(Tr,Ts,F\right)*P_{s}O_{2}\left(Ts\right)$$

where *Corr* is a correction factor for the oxygen measure, Tr is the reference temperature, usually 37 °C, Ts is the skin temperature, F is the correction factor to account for the flow, and P_s_O_2_ (Ts) is the skin oxygen partial pressure.

It is also specified that, considering the blood flow, F is relevant when the CO_2_ blood flow is small; the same applies to O_2_. This highlights the important outcome that concentrations of blood gases can be reliably and safely measured also in patients with circulatory disorders and changing blood flow. The device also comprises a pulse-oximetric unit for the measurement of oxygen saturation, which can be used for tissue blood flow F measurements as well.

A device presented in 2019 named OxiVenT™ (Sentec AG, Cambridge, UK, ref. [10]) is one of the possible sensors connected to a SenTec Digital Monitor, and the only one other than a two-wavelength reflectance pulse oximetry unit and a Stow–Severinghaus-type PCO_2_ unit that also includes a PO_2_ unit [37,96]. The PO_2_ unit is based on luminescence quenching, designed to eliminate drift [10], improving the usability of PtO_2_ measurements. Moreover, the two optical measurements, pulse oximetry and oxygen quenching, show mutual interference, even though the respective light sources are emitted alternatively to separate the two measurements. As for the OxiCarbo^®^ sensor, the authors mention how the presence of heating, envisaged for the electrochemical sensor, positively influences the SNR of the pulse oximetry measurement. The statistical analysis carried out for this sensor on the dynamic drift characteristic was conducted by exposing the sensor to a humified test gas for the calibration interval period (24 h for PtO_2_ and 12 h for PtCO_2_) and resulted in the PtO_2_ optical sensing unit providing a more accurate measurement from the onset than the electrochemical sensing portion, which, in addition, requires frequent calibrations, re-membraning, and the replacement of the electrolyte every 28–42 h. In 2022, this sensor was demonstrated to provide an accurate estimation of PtCO_2_ during general anesthesia in children [97].

### 6.3. O_2_/CO_2_ Concentrations

In 2020, Marasco et al. realized a non-invasive system [98] able to measure blood carbon dioxide and oxygen levels, based on optical sensors used in reflectance and transmission modes. The final purpose is to estimate the metabolic rate of the user and provide them with discrete and/or continuous metabolic rate measurements. The device is wearable, portable, or handheld and the system could also be incorporated into a pulse-oximeter. The sensing element is suggested to be put on a peripheral area of the body to reduce the complexity of the measurements.

Two acquisitions sets are taken. The first indicates the values of total blood oxygen content, obtained with a visible and near-infrared photodiodes; secondly, the values of the total blood carbon dioxide concentration in the blood are obtained using mid-infrared photodiodes.

For electronic circuits, O_2_ sensing necessitates a sample and hold circuit, whereas CO_2_ sensing needs a lock-in amplifier.

The sample and hold can work as a filter to provide a cleaner output, not affected by the photodiodes’ rapid change in signals.

The lock-in amplifier comprises a modulator and demodulator circuit used to filter noise of the signal, so as to increase the signal to noise ratio.

In tissues, in the mid-infrared range, water absorbance can become prevalent [99] and cause a reduction in the SNR. This would explain the presence of the lock-in, as suggested by Dervieux et al. [34].

## 7. Discussion

The Clark’s electrode and the Severinghaus electrode are the gold standard for measurements, respectively, of transcutaneous O_2_ and CO_2_ and are still widely used in clinical applications and in combination with other techniques, such as pulse oximetry. However, due to the limitations of these sensors, such as their bulky instrumentation, further studies on the same working principles have been conducted, both for O_2_ and CO_2_. Anyway, the intrinsic limitations of electrochemical technology have not been overcome, prompting a shift in research focus towards alternative technologies [34].

Furthermore, the need for continuous monitoring, particularly beyond clinical settings, has directed attention towards wearable and implantable technologies. Wearable integrated sensors can be attached to a patient’s body, thus representing a more comfortable device for the patient and allowing for a reduction in hospitalization, which is also reflected in cost reduction [53].

To pursue this aim, other technologies, different from electrochemical ones, have been developed.

Among them, luminescence-based sensors, with their low cost and flexibility, have gained a central role for both O_2_ and CO_2_ measurements. They have been shown to be fast (1.5 times faster than commercially available CO_2_ sensors, according to [60]) and designable to detect very low and high concentrations or partial pressures [56]. Moreover, they do not require a heating system, even if this is reflected in a reduction in the diffusion rate and a worse SNR.

Although luminescence-based sensors have turned out to be the most promising ones, other techniques have been investigated.

For CO_2_, the non-dispersive infrared (NDIR) principle is the most preferable transcutaneous gas detection system [89], since it is very accurate and able to reach low levels of detection, thanks to the specific absorbance peak of CO_2_. In addition, applying a PDMS membrane to sensors or using dual-wavelength NDIR optical sensors and then performing a differential measurement [69] are common solutions to avoiding the interference of water vapor affecting the signal, also improving the response time, which is in the order of minutes, and lifetime [40].

Furthermore, according to the literature [1,15], the rate-based method would be a valid solution for CO_2_ measurements to be implemented in wearable devices, even if, at present, they are too bulky to be included in a wearable device.

Finally, regarding the most recent technologies, electronic paramagnetic resonance (EPR) and photoacoustic sensors are being developed and are very innovative, since they are based on methods usually not employed for transcutaneous monitoring. The former exploits the electromagnetic properties of oxygen and has turned out to be an effective way to implement a thin and small sensor to detect very low levels of oxygen; however, its sensitivity depends on temperature [7]. The latter, for CO_2_ monitoring, consists of a quartz-enhanced photoacoustic spectroscopy (QEPAS)-based sensor [70], validated as a long-term stable, sensitive, and selective gas detection system. Even if heating up to 42–44 °C is not needed, specific solutions must be implemented to avoid the interference of environmental conditions, in which temperature is included, on the output signal.

Concerning miniature implantable sensors, the technologies proposed in this review represent a different innovative frontier regarding the measurement of O_2_. Indeed, they can be used to assess several clinical conditions, such as hypoxic regions in a tumor, ref. [71], which makes these types of sensors suitable for other contexts of application with respect to wearable transcutaneous (non-invasive) devices. However, wearable technologies can count on several advantages, among which, they are non-invasive and user-friendly and may be applied by an operator or by the patients themselves. On the contrary, implantable sensors must be inserted into the patient surgically by a specialist, with consequent risks of infection. Moreover, there are strict criteria for the fabrication process to guarantee biocompatibility and prevent biofouling [71].

## 8. Conclusions

In conclusion, the assessment of a patient’s respiratory status through the continuous monitoring of the partial pressures of oxygen and carbon dioxide is crucial. The gold standard, ABG analysis, while accurate, is limited by its painful nature and inability to provide continuous parameter assessment, a constraint particularly noteworthy in scenarios with significant and rapid parameter variations, such as in mechanically ventilated patients.

To address the need for the comprehensive, accurate, and continuous monitoring of arterial blood oxygen and carbon dioxide levels, transcutaneous monitoring has been proposed. Developed to meet the demands of NICU and ICU settings, transcutaneous monitoring not only provides information on ventilation quality, but also facilitates the early detection of pathological conditions related to tissue perfusion.

Despite the prevalent use of electrochemical sensors, mainly based on the Clark and Severinghaus electrodes, their limitations, including continuous calibration, heating requirements, and the need for expertise, have spurred the exploration of alternative working principles and sensor technologies. The current emphasis is on the development of wearable and easily deployable devices for remote patient monitoring outside clinical settings.

Our research suggests promising outcomes from various technologies, with optical methods based on luminescence quenching emerging as particularly encouraging.

Future research endeavors may involve extensive testing across diverse populations and the potential incorporation of mathematical models or artificial intelligence to enhance accuracy and reliability, customizing devices for individualized applications. In fact, in a study about the correlation between PtCO_2_ and PaCO_2_ [23], the authors suggested a multiple regression model to correct drifts over time in PtCO_2_ measurements during exercise. Another study suggested the use of AI to interpret the spectral data in the field of SpO_2_ measurements, accounting for potential confounding factors such as the volume fraction of melanosomes [100].

In conclusion, the exploration of new technologies for transcutaneous monitoring represents a promising research field, aiming to achieve measurement accuracy and reliability comparable to gold-standard technologies.

## Data Availability

No new data were created or analyzed in this study. Data sharing is not applicable to this article.
